# Genome and transcriptome analysis of surfactin biosynthesis in *Bacillus amyloliquefaciens* MT45

**DOI:** 10.1038/srep40976

**Published:** 2017-01-23

**Authors:** Yan Zhi, Qun Wu, Yan Xu

**Affiliations:** 1 State Key Laboratory of Food Science and Technology, Jiangnan University, Wuxi, 214122, China; 2 The Key Laboratory of Industrial Biotechnology, Ministry of Education, Jiangnan University, Wuxi, 214122, China; 3 Synergetic Innovation Centre of Food Safety and Nutrition, Jiangnan University, Wuxi, 214122, China; 4 School of Biotechnology, Jiangnan University, Wuxi, 214122, China

## Abstract

Natural *Bacillus* isolates generate limited amounts of surfactin (<10% of their biomass), which functions as an antibiotic or signalling molecule in inter-/intra-specific interactions. However, overproduction of surfactin in *Bacillus amyloliquefaciens* MT45 was observed at a titre of 2.93 g/l, which is equivalent to half of the maximum biomass. To systemically unravel this efficient biosynthetic process, the genome and transcriptome of this bacterium were compared with those of *B. amyloliquefaciens* type strain DSM7^T^. MT45 possesses a smaller genome while containing more unique transporters and resistance-associated genes. Comparative transcriptome analysis revealed notable enrichment of the surfactin synthesis pathway in MT45, including central carbon metabolism and fatty acid biosynthesis to provide sufficient quantities of building precursors. Most importantly, the modular surfactin synthase overexpressed (9 to 49-fold) in MT45 compared to DSM7^T^ suggested efficient surfactin assembly and resulted in the overproduction of surfactin. Furthermore, based on the expression trends observed in the transcriptome, there are multiple potential regulatory genes mediating the expression of surfactin synthase. Thus, the results of the present study provide new insights regarding the synthesis and regulation of surfactin in high-producing strain and enrich the genomic and transcriptomic resources available for *B. amyloliquefaciens*.

Surfactin is a lipopeptide that is synthesized by non-ribosome peptide synthases (NRPS) encoded by the *srfA* operon (*srfAA, srfAB, srfAC*, and *srfAD*)^1^. Surfactin comprises a long chain fatty acid tail and a circular heptapeptide containing glutamate acid (Glu), aspartate acid (Asp), valine (Val), and leucine (Leu). Surfactin has been recognized as one of the most promising biosurfactants and has broad application prospects in various industrial fields, including oil recovery, biopesticides, food processing, cosmetic, and pharmaceuticals, reflecting its unique properties, i.e., lower toxicity, higher biodegradability, and effectiveness under extreme conditions^2^. However, a significant obstacle for the large-scale application of surfactin is its low product yield per cell and the resulting high production costs.

The low yield of surfactin may be attributable to its biosynthetic regulation by a quorum sensing system, in which surfactin synthesis, competence development and sporulation are cross-linked within a complex network of pheromones and pleiotropic regulators. *Bacillus* cells constantly secrete ComX pheromone into the medium with increasing cell density^1^. The membrane receptor ComP senses ComX at a critical concentration and autophosphorylates and activates the cognate response regulator ComA. Subsequently, phosphorylated ComA (ComA-P) activates the transcription of the *srfA* operon after binding to the promoter region. The concentration of ComA-P in the cytoplasm is strongly influenced by several Rap-Phr family regulators, and the expression of the *srfA* operon is also regulated through several global regulators, including DegU, AbrB, and CodY^3^,^4^,^5^. As a consequence of quorum sensing, surfactin synthesis is dependent on cell density, preventing constant production and limiting overall yields.

In addition to this complex regulation mechanism, only a subpopulation (approximately 10%) of *Bacillus subtilis* cells sense the ComX pheromone and initiate surfactin production^6^, which may also reflect the low productivity of surfactin. Surfactin, secreted into the medium, acts as an extracellular signal that triggers another subpopulation of *Bacillus* cells to conduct cellular differentiation through the production of extracellular matrix^6^. During prolonged growth, matrix-producing cells undergo sporulation^7^. The matrix opportunely suppresses surfactin production by blocking the interaction between ComX and ComP without interfering with ComX production^6^. Therefore, once surfactin-responsive cells commence matrix production, these cells no longer respond to ComX, and no additional cells become surfactin producers^6^. This mechanism would reasonably explain why most *Bacillus* species exhibit limited surfactin synthesis.

Surfactin has been also described to act as an antibiotic during interspecific interactions between *Bacillus* and other species in the same ecosystem through the disruption or disintegration of cell membranes via physicochemical interactions^8^,^9^. For instance, surfactin produced by *Bacillus* sp. H2O-1 was reported to inhibit the growth of sulfate reducing bacteria^10^; Leu^7^-surfactin from *Bacillus mojavensis* showed antibiotic activity against *Fusarium verticillioides*^11^; and surfactin from *B. amyloliquefaciens* 1–45 showed antibacterial activity against *Streptomyces* species^12^. When the surfactin concentration is below or near the critical micelle concentration (approximately 0.01–0.025 g/L)^13^,^14^, surfactin monomers insert into phospholipid layers in biomimetic membrane systems, inducing mild content leakage^15^. In contrast, at higher concentrations, surfactin further attacks the phospholipid bi-layer, resulting in membrane solubilisation and vesicle destruction^8^,^9^. This mechanism may represent a third reason why most surfactin producers are unable to produce and secrete this antibiotic at high concentrations.

Because low productivity largely limits the commercial application of surfactin^2^, numerous efforts have been made in recent decades to improve surfactin production by optimizing the fermentation process^16^,^17^,^18^,^19^,^20^. However, these efforts have not yet successfully generated commercially viable and profitable surfactin production and will be unable to do so unless the yield of the final product from producer organisms is naturally high^2^. Therefore, surfactin producers demonstrating enhanced productivity per cell were constructed after screening for overproducing mutants or establishing genetically modified strains^21^,^22^. For example, recombinant strains characterized by promoter exchange of the *srfA* operon were constructed to eliminate surfactin synthesis through quorum sensing and constitutively produce surfactin^23^. Unfortunately, these attempts have met with limited success, and it remains difficult to meet the needs of industrial applications, reflecting the complex intrinsic regulatory network underlying *srfA* expression^22^,^23^.

In the present study, a *Bacillus amyloliquefaciens* strain (MT45) producing high levels of surfactin was obtained. Phenotype characterization indicated vigorous surfactin production of this strain independent of cell density, and the high surfactin concentration observed in the culture media provided evidence of the remarkable self-resistance capacity of this bacterium. Therefore, we sought to systemically unravel the biosynthesis and regulatory features underlying the overproduction of surfactin exhibited by this strain. Genome sequencing in combination with global transcriptome analysis was employed, and the results were compared with *B. amyloliquefaciens* type strain DSM7^T^, which produces much less surfactin. These results will allow us to better understand the mechanisms underlying surfactin overproduction and provide valuable information regarding potential molecular targets for rational strain improvement.

## Results and Discussion

### Phenotypic assays for biomass and surfactin production by MT45

**Figure 1:**
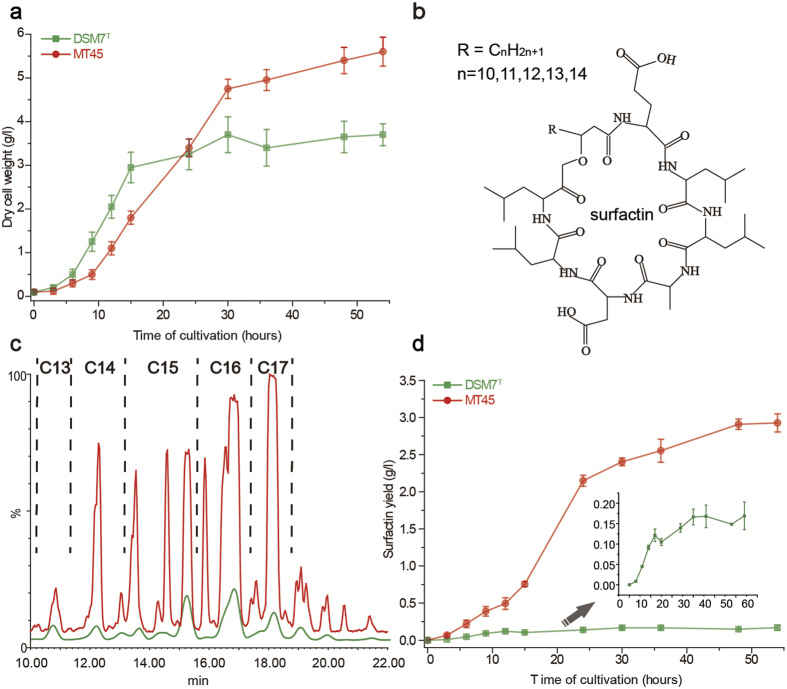
Cell growth and surfactin production of *B. amyloliquefaciens* MT45 and DSM7^T^. (**a**) Growth curve and (**d**) surfactin production curve in MMS media throughout the cultivation period. (**b**) Chemical structure of surfactin. (**c**) Total ion chromatogram of crude surfactin extracted from the culture broth of MT45 and DSM7^T^.

*Bacillus* cells were cultivated and regularly sampled. MT45 grew more slowly during the first 24 h and exhibited a longer logarithmic phase than DSM7^T^ (Fig. 1a). The biomass of MT45 steadily increased through the end of cultivation, reaching a maximum value of 5.6 g/l at 54 h. In contrast, the biomass of DSM7^T^ reached 3.7 g/l at 30 h and remained relatively stable during prolonged fermentation. Surfactin in the culture broth was characterized by LC-MS with standard surfactin as reference. MT45 and DSM7^T^ produced similar surfactin mixtures, comprising homologues with [M + H]^+^ peaks at 1008.68, 1022.68, 1036.70, 1050.68, and 1064.70, consistent with standard surfactin isomers with fatty acid chains of different lengths (C13, C14, C15, C16, and C17, respectively) (Fig. 1c)^10^.

The MS/MS fragments of surfactin produced in this study agreed with the surfactin standard (Supplementary Fig. S1) and suggested the sequence as Glu-Leu-Leu-Val-Asp-Leu-Leu. In addition, the retention times of surfactin from MT45 were identical to the standard surfactin from Sigma (Supplementary Fig. S2). Thus surfactins and their homologues were quantified by ultra-high performance liquid chromatogram (UPLC), and the amounts of all homologues were summed to obtain the total surfactin concentration. As shown in Fig. 1d, the maximum concentration of surfactin produced by DSM7^T^ at 36 h was 0.17 g/l, consistent with previous reports describing the production of limited surfactin by most *Bacillus* strains to act as a signalling molecule in intracellular interactions^24^,^25^. In contrast, surfactin produced by MT45 reached a maximum concentration of 2.93 g/l at 48 h and maintained stable levels during cultivation thereafter; thus, MT45 significantly differs from DSM7^T^ in terms of surfactin productivity. Additionally, the high surfactin concentration (>100-fold of CMC) in the culture broth indicated the strong antibiotic resistance capacity of MT45. The specific production rate (*q*_p_) of surfactin was calculated, and the maximum *q*_p_ (g _product_/g _dry cell weight_ •h) values for MT45 and DSM7^T^ were 0.16 and 0.04, respectively. The efficient production of surfactin by MT45 reflects high metabolic activity rather than high bacterial biomass.

In addition to surfactin, MT45 also produced iturin family lipopeptides with [M + H]^+^ peaks at 1031.76, 1045.78, 1059.78 (Supplementary Fig. S3), which is identical to bacillomycin D^26^. However, the concentration of bacillomycin D is much lower than that of surfactin since the peak area of bacillomycin D is only about 3.6% of that of surfactin in the total ion chromatograms. In addition, bacillomycin D was not detected in the culture broth of DSM7^T^. Neither MT45 nor DSM7^T^ produced fengycin family lipopeptides.

### Genomes and comparative genomic analysis

**Figure 2:**
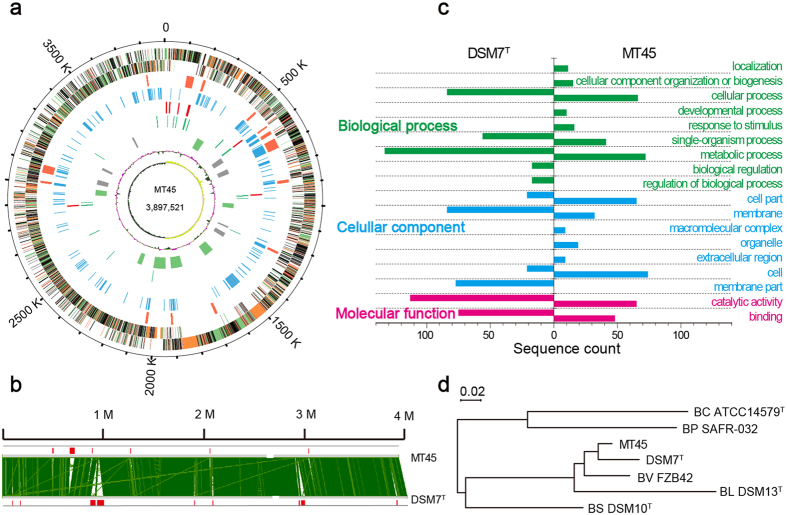
Genomic and comparative genomic analyses. (**a**) Circular representation of the MT45 genome. From the outside inwards, circle 1: scale; circles 2 and 3: the predicted CDSs colour-coded according to their functions: cellular process, green; metabolism, pink; information pathway, orange; other functions, red; unknown, black; circle 4: predicted genomic islands; circle 5: unique genes in MT45; circle 6: rRNA; circle 7: tRNA; circle 8: predicted prophages (grey) and gene clusters involved in secondary metabolite synthesis (green); and circles 9 and 10: GC content and GC skew (G+C/G−C), respectively. (**b**) Global alignment of the bacterial chromosomes constructed using the M-GCAT programme; indels are depicted as red rectangles. (**c**) GO classification of the unique genes in MT45 and DSM7^T^. (**d**) Phylogenetic tree generated via comparison of the genomes of *B. amyloliquefaciens* MT45, *B. amyloliquefaciens* DSM7^T^, *B. velezensis* FZB42 (BV), *B. subtilis* DSM10^T^ (BS), *B. licheniformis* DSM13^T^ (BL), *B. pumilus* SAFR-032 (BP), and *B. cereus* ATCC14579^T^ (BC).

To investigate the efficient biosynthesis features of surfactin in MT45, the entire genome was sequenced, revealing a complete circular genome 3,897,521 bp in length with a GC content of 46.09%. The genome of MT45 is smaller than those of most *Bacillus* species deposited in the NCBI database (Table 1). The principal features of the MT45 genome are shown in Fig. 2a, in the form of a circular graph. To detect the evolutionary origin of MT45, a neighbour-joining tree was constructed based on multi-genome alignment, including MT45 and the representative genomes of 6 other species in Table 1. The phylogram in Fig. 2d suggests a closer relationship between MT45 and DSM7^T^.

Given the enormous differences in surfactin productivity between MT45 and DSM7^T^, a global alignment of the two bacterial chromosomes was constructed using M-GCAT in combination with Mauve software. As shown in Fig. 2b, three large insertions (longer than 30 kb, depicted as red rectangles) were uniquely observed in the genome of DSM7^T^, primarily comprising phage-related integrase/recombinase and hypothetical proteins (Supplementary dataset 1). In addition, many unique regions in MT45 were annotated as transporters and antimicrobial peptide resistance-associated genes, separate from phage-related proteins (Supplementary dataset 2). These unique transporters and resistance proteins may contribute to the self-resistance of MT45 against high concentrations of antimicrobial surfactin. According to the pairwise genome comparisons of MT45 and DSM7^T^, there were 332 and 562 singletons in MT45 and DSM7^T^, respectively. These sequences were further annotated and analysed using the Blast2Go programme. A total of 298 and 399 unique sequences for MT45 and DSM7^T^, respectively, were assigned to biological processes, cellular components, and molecular functions (Fig. 2c, Supplementary dataset 3 and 4).

In the biological process category, which is closely associated with cell metabolism activity, 52 singletons in MT45 were uniquely categorized as 4 secondary gene ontology (GO) items, including localization (11 genes), cellular component organization or biogenesis (15 genes), developmental process (10 genes), and response to stimulus (16 genes). Amongst these genes, WV34_01080, WV34_07040, and WV34_04495, categorized under localization, were annotated as ATP-binding cassette (ABC) transporters. Previous studies have implicated ABC transporters in the secretion of antibiotics through the cell membrane, contributing to bacterial self-resistance^27^. These proteins may assist MT45 in the secretion and resistance of high concentrations of surfactin. In addition, an 8,708-bp gene cluster containing WV34_01100, WV34_01105, WV34_01120, and WV34_01125, categorized under response to stimulus, was annotated as an antimicrobial peptide transporter. This unique gene cluster may be involved in surfactin resistance, as WV34_01125 shares high amino acid sequence identity with SwrC, a membrane protein responsible for the secretion and self-resistance of surfactin^28^.

To better establish the uniqueness of the regions in strain MT45, a broader comparative genome analysis was performed by comparing the genome of MT45 with 17 other genomes of *B. amyloliquefaciens* strains (Supplementary Table S1). The phylogram shown in Supplementary Fig. S4 suggested a higher genomic distance of MT45 with other strains. A total of 183 genes were found to be unique in the genome of MT45 (Supplementary dataset 2), including 8 genes encoding ABC transporters and 2 resistance-related genes (WV34_01120 and WV34_01125) which were supposed to be potentially associated with surfactin resistance as mentioned above.

### Global transcriptome analysis

**Figure 3:**
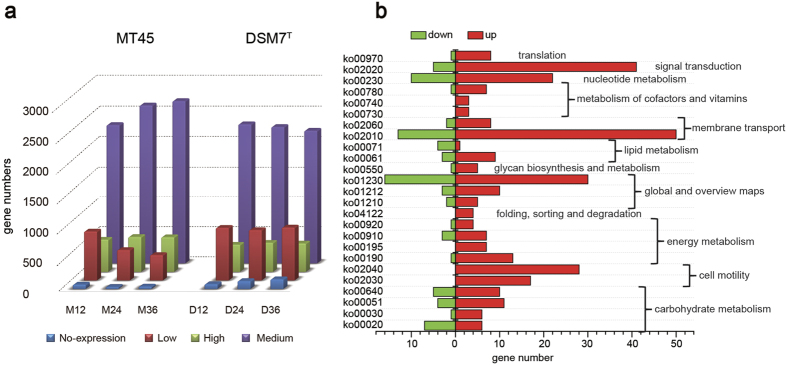
Transcriptomic and comparative transcriptomic features of *B. amyloliquefaciens* MT45 and DSM7^T^. (**a**) Global abundance of the gene expression of MT45 and DSM7^T^ throughout the cultivation period. The transcripts were assessed based on FPKM values: high expression (FPKM ≥ 500), medium expression (10 ≤ FPKM < 500), low expression (1 ≤ FPKM < 10), and non-expression (FPKM < 1). (**b**) Major differences in metabolic pathways of MT45 compared with DSM7^T^ cultivated for 12 h.

RNA-Seq was employed to reveal the transcriptomic features of overproduction of surfactin in MT45 during different growth stages (12, 24, and 36 h), with DSM7^T^ as a comparative control. For simplicity, the transcriptomes obtained at specific time points (in h) were designated M12, M24, and M36 for MT45 and D12, D24, and D36 for DSM7^T^. More than ten million reads for each sample were generated with clean ratios >82%. The clean reads generated average mapping ratios of 92.87% and 99.47% for the genomes of MT45 and DSM7^T^, respectively. The mapped genes were classified into 4 groups based on the expression levels indicated by FPKM (Fragments Per Kilobase of exon model per Million mapped Reads) values (Fig. 3a). Using a threshold of FPKM >1 to define potential gene expression, averages of 3,677 and 3,586 expressed genes were observed for MT45 and DSM7^T^, respectively. Although most genes were medially expressed in both MT45 and DSM7^T^ throughout cultivation, genes expressed at high levels in MT45 were more numerous than those in DSM7^T^ during the 3 different growth stages. The average number of highly expressed genes in MT45 throughout cultivation was 563, while an average of 467 genes were highly expressed in DSM7^T^. In addition, the average numbers of non-expression and low-expression genes in MT45 were 48 and 578, respectively, which were much lower than those in DSM7^T^, which had 124 non-expression genes and 861 low-expression genes. Furthermore, the number of non-expression genes in MT45 decreased from 12 to 36 h, in contrast with DSM7^T^, for which the number of non-expression genes increased over time. This result was consistent with the large numbers of phage-related and/or hypothetical regions in the genome of DSM7^T^, which are silenced during cultivation (Supplementary dataset 1). Therefore, a smaller genome with less nonessential genes may benefit MT45 with accumulation of more biomass and bioproducts^29^,^30^.

Real-time PCR was performed on the original RNA extracts to confirm the transcriptional profiling data obtained from RNA sequencing. Although there were some differences in the fold-changes for several differentially expressed genes (DEGs) as determined by real-time qPCR and RNA-Seq, the general trends were consistent, supporting the credibility of the RNA-Seq data (Supplementary Table S2). These differences likely result from the use of different methods, a phenomenon that was also observed in previous studies^31^,^32^.

To further unravel the influence of transcriptional properties on the metabolic activity of MT45 and DSM7^T^, a comparative transcriptome analysis was performed at 12 h when specific surfactin production rates were at the maximum values. The distribution of DEGs in metabolic pathways was examined by employing KEGG (Kyoto Encyclopaedia of Genes and Genomes) enrichment. Twenty-five most respondent pathways (*P* < 0.05) were assigned and categorized into 12 items, comprising 315 up-regulated genes and 83 down-regulated genes (Fig. 3b). The KO numbers and the corresponding pathways are shown in Supplementary dataset 5. Amongst these pathways, signal transduction pathway (two-component system) and membrane transport pathway (including ABC transporters and the phosphotransferase system) contain the greatest number of up-regulated genes.

The two-component system (TCS) is an important signal transduction system comprising a sensor kinase and a response regulator. Bacteria heavily rely on the TCS to detect environmental signals and process information^33^. Among the DEGs annotated as components of the TCS, the *narGHJI* operon, involved in nitrogen metabolism, was overexpressed in MT45 with an average FPKM of 1,319.8 but was not expressed in DSM7^T^. The *narGHJI* operon is induced under oxygen-limited conditions through Fnr, which is required for the adaptation of *B. subtilis* to low oxygen^34^. *B. subtilis* is able to grow anaerobically via respiration with nitrate as a terminal electron acceptor^35^. The utilization of nitrate likely occurred in the present study following the depletion of ammonium in the media, with subsequent induction of dissimilatory nitrate metabolism. Therefore, the high expression of *narGHJI* in MT45 may result in the efficient utilization of nitrogen sources, thus contributing to high surfactin yields. These results are consistent with those of a previous report in which surfactin production in *B. subtilis* ATCC21332 was strongly enhanced under nitrate metabolism^36^.

Among the significantly responsive ABC transporters, the *glnQHMP* operon was assigned as a glutamate transporter. The average FPKM value of *glnQHMP* in MT45 was 541.2, but it was not expressed in DSM7^T^. Glutamate is the first amino acid of the heptapeptide sequence in surfactin, and the synthesis of surfactin is initiated from the condensation of glutamate and fatty acid^37^. High-level glutamate transporter operon expression may provide MT45 with more glutamate for the efficient synthesis of surfactin. More importantly, ABC transporters together with the neighbouring TCS have been recognized as detoxification systems for resistance against antimicrobial peptides in many Firmicutes bacteria^38^,^39^. Surfactin works as an antibiotic by attacking the bacterial cell membrane^9^. Up-regulated ABC transporters are important for the efflux and survival of MT45 in the presence of high concentrations of surfactin^9^.

Other DEGs were primarily enriched in the citrate cycle, the pentose phosphate pathway, fructose and mannose metabolism, and lipid metabolism. These processes provide essential precursors, such as amino acids, carbon skeleton, NADPH, and ATP, for cell growth and surfactin biosynthesis. Therefore, it is important to analyse these pathways in detail to obtain a better understanding of the high efficiency of surfactin biosynthesis.

### Construction of a KEGG pathway map for surfactin biosynthesis

**Figure 4:**
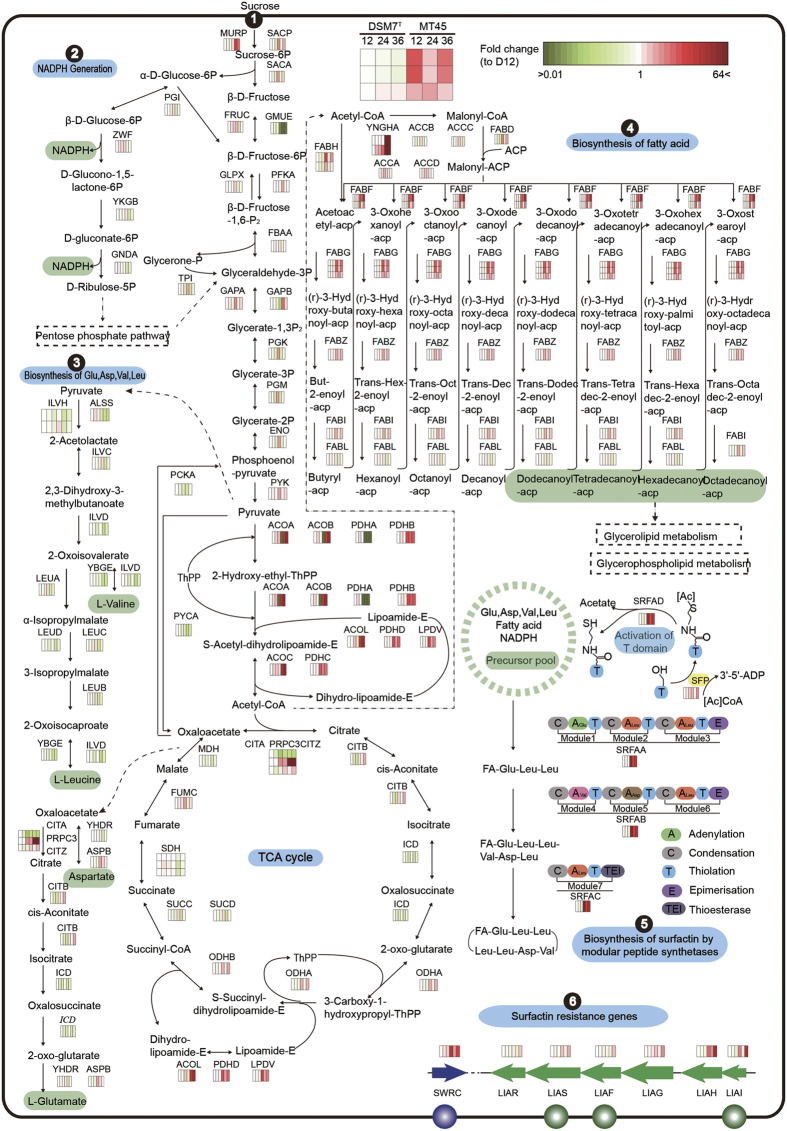
Metabolic pathway map for surfactin biosynthesis. The metabolic network was reconstructed based on KEGG pathway analysis. Six modules were partitioned according to function. Transcripts of MT45 and DSM7^T^ are shown near the pathway as a heat map, based on fold-changes in transcript levels relative to DSM7^T^ after 12 h. Colour legend is shown at the top left of the map. Gene abbreviations are shown in Supplementary dataset 6.

A global surfactin biosynthesis pathway in *B. amyloliquefaciens* was constructed based on the reported surfactin production process in *B. subtilis*, as *B. amyloliquefaciens* and *B. subtilis* share many common properties only with hydrolases production as major recognized difference^40^. Levels of individual transcripts were expressed in a heat map and subsequently applied to a metabolic pathway map (Fig. 4, Supplementary dataset 6). Six modules were partitioned according to their functions, specifically glycolysis and tricarboxylic acid^41^ cycle, NADPH generation, amino acids biosynthesis, fatty acid biosynthesis, modularly enzymatic synthesis of surfactin, and secretion and resistance of surfactin.

Module 1 of the pathway represented sucrose utilization and the subsequent glycolysis pathway and TCA cycle, which provide building precursors for cellular metabolism. The genes involved in the utilization of sucrose, including *sacP, murP* and *sacA*, which encode a sugar transporter, permease, and sucrose-6-phosphate hydrolase, respectively, were highly expressed in MT45. Most of the genes involved in glycolysis and the TCA cycle were also up-regulated in MT45, providing carbon skeleton and energy for subsequent metabolism. For example, pyruvate, 2-oxo-glutarate, oxaloacetate, and acetyl-CoA serve as building precursors for the biosynthesis of Val/Leu, Glu/Asp, and fatty acids, respectively.

Module 2 comprised a portion of the pentose phosphate pathway, which primarily generates NADPH and pentose for cell metabolism. ZWF and GNDA, which catalyse the generation of NADPH, were highly expressed in MT45 at 12 h but were not significantly different, suggesting NADPH is not the bottleneck for surfactin production. Module 3 involved the biosynthesis of Glu, Asp, Val, and Leu, which are intrinsic components of surfactin. Higher expression levels of aspartate aminotransferase (AspB and YhdR) in MT45 promote the synthesis of Glu/Asp and subsequent surfactin assembly. In addition, genes for the synthesis of Val and Leu were not expressed as highly in MT45, consistent with a previous report in which *srfA* expression was negatively regulated through the metabolism of branched chain amino acids^3^. Fatty acids are another intrinsic component of surfactin, comprising the hydrophobic tail. Most genes in the fatty acid synthesis pathway shown in module 4 were up-regulated in MT45. The biosynthesis of fatty acids is initiated from the building precursor acetyl-CoA. Notably, the genes that participate in acetyl-CoA generation were particularly up-regulated in MT45, which would be highly beneficial for the biosynthesis of fatty acids. The increased fatty acid biosynthesis pathway is likely essential for highly efficient surfactin production. Increased fatty acid biosynthesis may also explain the higher biomass of MT45.

The biosynthesis of surfactin is catalysed through NRPS, initiated from the condensation of fatty acids and Glu. Other constituent amino acids are assembled through the NRPS multi-enzyme complex, comprising adenylation, condensation, and thiolation domains responsible for the activation of amino acids and peptide chain elongation^42^. As shown in module 5, the *srfA* operon was highly expressed in MT45 (9.25–48.86-fold compared with DSM7^T^). Overexpression of the surfactin synthase gene would be closely associated with the high surfactin productivity of MT45.

Amino acids and fatty acids in the cytoplasm are generally utilized to synthesize cell structural elements and various enzymes. Therefore, surfactin biosynthesis inevitably competes with biomass formation. Aminoacyl tRNA synthases play important roles in protein translation. Thus, the relative expression levels of the aminoacyl-tRNA synthases and *srfA* operon reflect competition for the utilization of amino acids for cell growth or surfactin production. In MT45, the FPKM value of *srfAA* was >2.99-fold higher than that of glutamyl-tRNA synthase (*gltX*) (Supplementary Fig. S5b,d), and the expression of *aspS* (aspartyl-tRNA synthase), *valS* (valyl-tRNA synthase), and *leuS* (leucyl-tRNA synthase) was lower than for the other genes of the *srfA* operon. In contrast, *gltX, leuS*, and *aspS* were highly expressed compared with the *srfA* operon in DSM7^T^ throughout cell growth. The overexpression of *aprE*, an extracellular alkaline serine protease with an average FPKM of 27,506.3, further supports the preferential usage of amino acids in the cytoplasm of DSM7^T^ for protein synthesis (Supplementary dataset 7). In addition, fatty acids are competitive substrates for the biosynthesis of glycerophospholipids, an essential component of the cell membrane. The expression of *srfAA* in MT45 was 2.78–13.9-fold higher than that of the genes that convert fatty acids and glycerone phosphate to phosphatidate (Supplementary Fig. S5c,e). Based on these results, additional amino acids and fatty acids may be used to produce surfactin and provide a reasonable explanation for the lower growth rate of MT45 prior to 24 h.

Module 6 comprises the expression of surfactin resistance genes. Reflecting antibiotic activity, self-resistance is essential for the high productivity of surfactin. SwrC, important for the export and self-resistance of surfactin in *B. subtilis*^15^, was highly expressed in MT45 with an overall FPKM value of 992.01, indicative of its significant effects on surfactin efflux. In addition, the *liaRSFGHI* operon, annotated as genes associated with the resistance of daptomycin (a structural analogue of surfactin), was significantly expressed in MT45, particularly for *liaH* and *liaI*, with an FPKM value >800 at 36 h (Supplementary dataset 7). The overexpression of *liaRSFGHI* operon during the late stage of fermentation may help MT45 confront the high concentrations of surfactin; however, this hypothesis requires further investigation. Furthermore, unique transport and resistance genes in the MT45 genome were all expressed (Supplementary dataset 2), supporting their potential antagonistic functions against a high surfactin concentration.

### Differentially expressed genes associated with *srfA* expression

**Figure 5:**
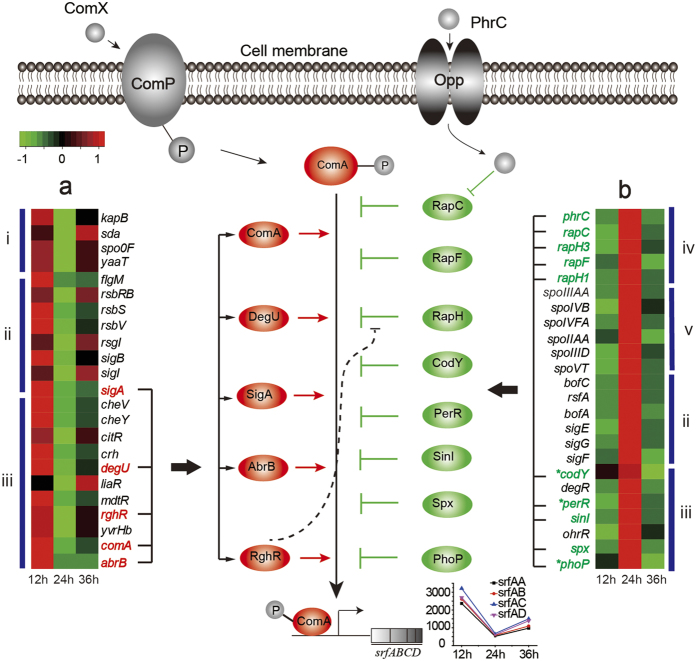
Schematic representation of the differentially expressed genes affecting *srfA* expression. Arrows indicate positive effects on *srfA* expression, the transformation of phosphoryl groups, or pentapeptide incorporation. T-bars indicate the negative effects on DNA binding or protein interactions. Bent arrow represents the promoter. ‘P’ in the circle represents the phosphoryl group. Heat maps in (**a**) and (**b**) indicate DEGs annotated as regulators that exhibit the same and opposing expression trends as *srfA*, respectively. Subgroups in (i)–(v) related to phosphorylation, sigma factors and their controls, transcription factors and their controls, genetic competence, and spore formation, respectively. Genes with fold-changes >2, detected by RT-qPCR, are indicated with asterisks.

The enormous difference in the surfactin yield between MT45 and DSM7^T^ is significantly influenced by the differential expression of the *srfA* operon. Therefore, it is necessary to study the regulatory mode of *srfA* expression in MT45. According to the transcriptome data, expression of *srfA* operon of MT45 decreased at 24 h and then subsequently increased. Generally, genes with the same or opposing expression tendency as *srfA* would be positively or negatively associated with *srfA* expression, respectively^4^. Therefore, DEGs exhibiting similar or opposing expression tendencies, were selected to further analyse their potential effect on *srfA* expression. Subsequently, the selected DEGs assigned the function of “regulation of gene expression” in the Subtiwiki database^43^ were further analysed, including 23 DEGs with similar expression tendencies to *srfA* and 24 DEGs with opposing expression tendencies (Fig. 5). These DEGs were categorized into 5 subgroups according to their functions: (i) phosphorylation, (ii) sigma factors and their controls, (iii) transcription factors and their controls, (iv) genetic competence, and (v) spore formation.

Among these 47 DEGs potentially associated with *srfA* expression, we found four positive regulators which is consistent with previous studies. First, as expected, *comA* and *sigA* were down-regulated at 24 h and subsequently up-regulated in the prolonged cultivation because ComA and SigA directly bind to the *srfA* promoter to initiate transcription. In addition, expression of *degU* in MT45 significantly decreased from 12 to 24 h with a fold-change >2.5 (*P* < 0.0001). *srfA* expression is reported to be influenced by DegU-P, as the disruption of *degU* decreases *srfA* expression in *B. subtilis*^44^. RghR represses the function of Rap proteins, which inhibit the binding of ComA and DegU to target promoters, leading to the inhibition of *srfA* expression^45^. In the present study, *rghR* exhibited the same expression tendency as *srfA* but an opposing expression tendency to RapG and RapH in MT45, suggesting its positive effects on *srfA* expression. The transcriptome data in combination with previous reports confirmed the dependability of the analytical method and the results of the present study.

The transition state regulator AbrB was the most significantly down-regulated gene at 24 h, with a fold-change >8.5 (*P* < 0.0001). This result was consistent with previous study in which *abrB* was highly expressed from the lag to the exponential phase but dynamically down-regulated when *Bacillus* cells entered the stationary phase^5^. *B. subtilis* expresses another protein, Abh, containing N-terminal DNA-binding domains highly homologous to those of AbrB^5^. The disruption of *abh* results in stronger *srfA* operon derepression than *abrB* deletion, indicating negative regulation of *srfA* expression by Abh^46^. However, AbrB binding to the *srfA* promoter in the *abh* deletion strain was retained, whereas Abh binding was weak on the *abrB* deletion background^46^. Therefore, the effects of *abh*–*abrB* on *sfrA* expression remain unclear. The expression of *abh* differed from *abrB* and remained relatively stable in MT45 throughout cultivation. Thus, AbrB, unlike Abh, may positively regulate *srfA* expression, reflecting its similar expression tendency as *srfA*, although the molecular mechanism remains unknown.

DEGs, which would be negatively associated with *srfA* expression of MT45, were shown in Fig. 5b. First, the transcriptome data suggested the suppression of *srfA* expression through multiple Rap proteins, i.e., RapH1, RapH3, and RapC, which are aspartate phosphatases controlling ComA activity and negatively influence surfactin production^45^. CodY was also negatively associated with *srfA* expression, consistent with the results of previous studies in which CodY repressed *srfA* expression through direct binding to the *srfA* promoter^46^,^47^. A recent study showed that deletion of *codY* could significantly improve the surfactin production in *B. subtilis*^48^, indicating that CodY is a negative regulator of *srfA*. Another potential repressor is Spx, which regulates gene expression in *B. subtilis* in response to oxidative stress through interactions with the C-terminal domain of RNA polymerase (RNAP) alpha subunit (α-CTD)^49^. RNAP interacts with the −35 region of *srfA*, assisted by ComA-P, to fully activate transcription. Furthermore, ComA-P interacts with the RNAP α-CTD in a region previously shown to interact with Spx^49^. Therefore, Spx suppresses *srfA* expression by blocking the interaction between ComA and RNAP in the promoter region via competition for an overlapping site in the α-CTD^49^. The up-regulated expression of SinI at 24 h is reasonable because SinI positively regulates matrix production, which suppresses the constitutive synthesis of surfactin^25^. Therefore, SinI negatively and indirectly regulates *srfA* expression.

According to the transcriptome and qPCR data, another potential repressor of *srfA* is PerR. This result differs from a previous report in which PerR positively regulated *sfrA* expression in *B. subtilis*^44^. The mechanism through which PerR affacts *srfA* remains unclear, but two PerR boxes were identified in the *srfA* promoter-distal region near the ComA box, indicating PerR may interact with ComA to influence the activation of *srfA* expression^44^. In addition, these disparities may reflect the use of different culture media and harvest time points^44^,^50^. Besides, *srfA* was reported recently to be positively regulated by PhoP under phosphate-limiting conditions in *B. subtilis*^51^. However, in the present study, *phoP* showed an opposing expression tendency to that of *srfA*, suggesting PhoP may be negatively associated with *srfA* expression under the culture conditions applied herein (phosphate enrichment).

Unlike MT45, the expression of *srfA* operon in DSM7^T^ continued to decline from 12 to 36 h (Supplementary Fig. S6). However, we obtained generally similar regulatory DEGs in DSM7^T^ with those in MT45. For instance, RghR and AbrB would positively regulated *srfA* expression since their expressions were down regulated across the cultivation, and RapC, RapF, RapH, PerR, SinR, and Spx would negatively regulated *srfA* expression since their expressions were continually up regulated. However, the effects of CodY, PhoP, and ComA on *srfA* expression is ambiguous in DSM7^T^, since their functions seem to be inconsistent with our hypothesis. The results obtained here further suggest that surfactin synthesis may be differentially regulated in minor and strong surfactin producers or in different *Bacillus* species and under different culture conditions^23^. Other DEGs (shown in Fig. 5) associated with competence development and spore formation also indicated the complexity of the transcriptional regulation of *srfA* and the cross-linked relationship between surfactin production and the two processes.

Therefore, in combination with the transcriptional data in this study and the regulators reported previously, we showed the potential genes and regulators that would be positively or negatively associated with *srfA* expression. Other DEGs, predicted based on transcriptional tendency, represent potential regulators and targets for the rational design of surfactin producers. In further studies, the function of these genes should be verified in a more comprehensive way, including gene expression and construction of gene deletion mutant, and applied to the artificial design of improving industrial surfactin production.

Overall, the results of the present study provide an integrated omics profile of a surfactin high-producing strain, facilitating the genomic and transcriptomic comparison of minor and strong surfactin producers, and reveal differences in *srfA* operon expression, precursor redirection, and antibiotic resistance capacity that significantly influence surfactin productivity. These findings will enrich the available genetic resources for surfactin-producing *Bacillus* and may provide fundamental information for future studies to artificially construct industrial strains via genetic modification or pathway engineering.

## Materials and Methods

### Microorganisms and growth conditions

*B. amyloliquefaciens* MT45 with high surfactin productivity was isolated from Chinese *Maotai Daqu* (Renhuai, China), and stored at China General Microbiology Culture Collection Centre (CGMCC: 12593). *B. amyloliquefaciens* type strain DSM7^T^ was obtained from DSMZ (German Collection of Microorganisms and Cell Cultures). *Bacillus* cells were cultivated at 30 °C in 500 ml flasks, containing 100 ml of minimum media (0.8% NH_4_NO_3_, 0.03 M KH_2_PO_4_, 0.04 M Na_2_HPO_4_, 7 μM CaCl_2_, 4 μM FeSO_4_, 100 μM MgSO_4_, 4 μM ethylene diamine tetraacetic acid) supplied with 4% sucrose (MMS media).

### Cell growth and surfactin production assays

To determine dry cell weight, a cell culture aliquot was sampled into a pre-weighed sampling tube and centrifuged at 8,000× *g* for 10 min. Subsequently, the supernatant was removed and the cell pellet was washed three times with sterile saline solution. The washed cell pellet was dried for at least 4 h to a constant weight at 105 °C. For surfactin determination, cell-free culture broth was adjusted to pH 2.0 using 6 M HCl and centrifuged at 10,000× *g* for 30 min. Crude surfactin was obtained after removing the supernatant and re-dissolving the pellet in 100% methanol. Surfactin composition and other potential lipopeptides were characterized by LC-MS as previously reported^12^. The quantitative analysis of surfactin was performed on Waters UPLC H-class system equipped with a binary solvent delivery system and an auto-sampler. The chromatographic separation was performed on a Waters Acquity C18 column (50 mm × 2.1 mm, 1.7 μm particle). Injection volume was 5 μL. The mobile phase consisted of solvent A (HPLC grade water containing 0.1% formic acid) and solvent B (HPLC grade methanol). Elution was performed by linear biphasic gradient of 85–100% solvent B over 6 min, at a flow rate of 0.3 mL/min. The elution pattern was monitored by determining absorbance at 215 nm. The concentration of surfactin was analysed and quantified by Empower Version 3.0, using a calibration curve made by surfactin standards (Sigma Chemicals, St. Louis, MO). Then the total surfactin concentration was calculated by summing up the concentration of each congener. All the experiments were performed in triplicate.

### Genomic sequencing and annotation

MT45 cells were cultivated for 24 h and subsequently harvested through centrifugation at 10,000× *g* for 5 min at 4 °C. Genomic DNA was immediately extracted as previously described^52^. The MT45 genome was sequenced using the whole-genome shotgun method on an Illumina Miseq platform. Four paired-end/mate-paired sequencing libraries were constructed with insert sizes of 450 bp, 700 bp, 3 kb, and 8 kb. The reads were assembled into contigs and scaffolds employing the *de novo* assembler Newbler^53^ and SSPACE^54^. Gaps were closed using the GapCloser programme^55^ combined with long-range PCR using Phusion polymerase from New England Biolabs Inc. (Beverly, MA, USA) and subsequent Sanger sequencing (Personalbio, Shanghai, China). The genome sequence of MT45 was deposited in GenBank under the Whole Genome Shotgun project [GenBank: CP011252].

The prediction of protein-encoding sequences was accomplished by employing both the MAKER pipeline prediction system and Glimmer 3^56^. Functional annotation was carried out using the BLASTP search tool with *B. amyloliquefaciens* DSM7^T^ and *B. subtilis* 168 as references, as well as the non-redundant protein database (nr) of GenBank (parameters: E-value: 1E-5, coverage >60%, identity >50%). Each gene was functionally classified into Cluster of Orthologous Genes (COG) categories by performing an RPS-BLAST search against the COG database with an E value of 1E-5^57^. Genes for tRNAs and rRNAs were predicted with tRNAscan-SE and RNAmmer 1.2 Server, respectively^58^,^59^. Horizontally transferred genomic islands (GIs) were identified with IslandViewer 3^60^, using Seq-Word Sniffer tools^61^ to combine the prediction results. Prophage regions were predicted using the PHAST server^62^. The circular map and the graphic representation of genome-compared orthologous genes were generated using Circos. Global alignment of whole genome sequences of MT45 and DSM7^T^ was performed using M-GCAT software^63^.

### Sequence comparison and construction of an evolutionary tree

Genomic comparison of MT45 and DSM7^T^ was performed using Mauve software, employing Progressive Mauve sequence alignment using default parameters. The resulting unique genes in MT45 and DSM7^T^ were functionally classified into GO categories using the Blast2Go programme. To construct an evolutionary tree, a family containing 7 closely related *Bacillus* genomes was generated using Orthomcl software to obtain the protein sequences with a strict 1:1:1 ratio. Subsequently, Muscle (version 3.8.31) was employed to compare the protein sequences using default parameters. No matching portions of the alignments were removed using Gblock (version 0.91 b). Finally, the phylogenetic tree was constructed using the PHYLIP package represented in Newick format.

### Transcriptomic analysis using RNA-seq

For RNA extraction, *Bacillus* cells were cultivated for 12, 24, and 36 h and harvested after centrifugation at 10,000× *g* for 5 min at 4 °C. The resulting pellets were immediately frozen in liquid nitrogen. Total RNA was extracted using an RNeasy Mini Kit (QIAGEN, GmBH, Germany) according to the manufacturer’s instructions. RNA quality was evaluated using an Agilent Bioanalyser 2100 system (Agilent Technologies, Santa Clara, CA, USA). Ribosomal RNAs were removed using the Ribo-Zero rRNA Removal Kit (Epicentre) for Gram-positive organisms prior to sequencing analysis. Subsequently, RNA was fragmented and used as a template for randomly primed PCR.

Strand-specific cDNA libraries were prepared employing standard techniques for subsequent Illumina sequencing using the mRNA-seq Sample Prep Kit (Illumina). The cDNA libraries were sequenced on an Illumina HiSeq 2500 according to the manufacturer’s instructions. Sequencing raw reads were pre-processed after filtering out rRNA reads, sequencing adapters, short-fragment reads and other low-quality reads. The remaining clear reads were mapped to the reference genome of MT45 or DSM7^T^ using Bowtie2 software based on the local alignment algorithm^64^. The expression levels were normalized to the library and the gene length by calculating the FPKM value^65^. Differential expression of all of transcripts was quantified using DESeq software^66^. The method of FDR (False Discovery Rate) control was used to correct the results for multiple hypothesis testing^67^. Significant DEGs were screened based on an FDR threshold of ≤ 0.001, and a |log_2_ Fold change| value ≥ 1. KOBAS software (KOBAS, Surrey, UK) was applied to test the statistical enrichment of DEGs in KEGG pathways using hypergeometric tests with a *P* value cut-off of 0.05. The RNA sequence data has been deposited to DDBJ (DNA Data Bank of Japan) with Accession ID DRA005269.

### Reconstruction of the KEGG pathway map

Functional annotation descriptions were assigned using BLASTP^68^ in conjunction with the KEGG database (E-value cut-off of 1E-10). A metabolic network was reconstructed using KEGG mapper (http://www.genome.jp/kegg/) with the KO numbers and gene abbreviations shown in Supplementary dataset 6. Subsequently, the KEGG map was manually redrawn using Adobe Illustrator CC 2014 (Adobe Systems). Heat maps of the expressed genes involved in surfactin biosynthesis were generated using the *heatmap.2* function from the *Gplots* package in *R* statistical software v. 3.1.0^69^. Statistical testing for gene expression was performed in R with DESeq using the no replicate method (Supplementary dataset 8)^66^.

### qRT - PCR assay

Real**-**time PCR was performed on the same RNA extracts using in RNA-seq to confirm the transcriptional data obtained from Illumina sequencing as well as to study the expression of *srfA* operon and its regulatory genes in MT45 and DSM7. Reverse transcription was performed using the QuantiNova Reverse Transcription Kit (QIAGEN, Hilden, Germany) with total RNA as template. The transcription levels of genes were detected by quantitative real-time PCR (qRT-PCR) with BIO-RAD CFX96 Touch q-PCR system and all regents for qPCR were from BIO-RAD Laboratories, Inc. (Hercules, CA, USA). The relative transcription levels of target genes were quantified by the 2^-ΔΔCT^ method using *recA* gene as an internal control. Each qPCR reaction was performed in 20 μL volume containing 10 μL of SsoFast EvaGreen Supermix, 0.4 μL of each primer, 1 μL of template cDNA and 8.2 μL of RNase-free H_2_O. The amplification conditions were as follows: preheating at 95 °C for 2 min and then 40 cycles of 95 °C for 5 s, 60 °C for 15 s. Three technical replicates were carried out for each target gene.

## Additional Information

**How to cite this article**: Zhi, Y. *et al*. Genome and transcriptome analysis of surfactin biosynthesis in *Bacillus amyloliquefaciens* MT45. *Sci. Rep.* **7**, 40976; doi: 10.1038/srep40976 (2017).

**Publisher's note:** Springer Nature remains neutral with regard to jurisdictional claims in published maps and institutional affiliations.

## Supplementary Material

Supplementary Information

Supplementary Dataset 1

Supplementary Dataset 2

Supplementary Dataset 3

Supplementary Dataset 4

Supplementary Dataset 5

Supplementary Dataset 6

Supplementary Dataset 7

Supplementary Dataset 8

## Figures and Tables

**Table 1:** Genomic features of *B. amyloliquefaciens* MT45 and comparison with other *Bacillus* species.

Strain	Genome size (bp)	GC content (mol%)	Protein-coding sequences	rRNA	tRNA
*B. amyloliquefaciens* MT45	3,897,521	46.09	3691	24	81
*B. amyloliquefaciens* DSM7^T^	3,980,199	46.08	3922	30	94
*B. velezensis* FZB42	3,918,589	46.49	3693	30	86
*B. subtilis* DSM10^T^	4,215,610	43.51	4188	30	87
*B. licheniformis* DSM13^T^	4,222,645	46.19	4172	21	72
*B. pumilus* SAFR-032	3,704,645	41.29	3607	21	70
*B. cereus* ATCC14579^T^	5,411,809	35.3	5210	39	108
