# Supplementary material for: Genome and transcriptome analysis of surfactin biosynthesis in Bacillus amyloliquefaciens MT45
Source: Sci Rep. 2017 Jan 23;7:40976. doi: 10.1038/srep40976 (PMC5256033; doi:10.1038/srep40976)
Supplement: Supplementary Information [file srep40976-s1.pdf]

Genome and transcriptome analysis of surfactin biosynthesis in *Bacillus amyloliquefaciens* MT45

Yan Zhi[1, 2, 3, 4], Qun Wu[1, 2, 3, 4,*], and Yan Xu[1, 2, 3, 4,*]

[1]State Key Laboratory of Food Science and Technology, Jiangnan University, Wuxi, 214122, China

[2]The Key Laboratory of Industrial Biotechnology, Ministry of Education, Jiangnan University, Wuxi, 214122, China

[3]Synergetic Innovation Center of Food Safety and Nutrition, Jiangnan University, Wuxi, 214122, China

[4]School of Biotechnology, Jiangnan University, Wuxi, 214122, China

*Corresponding: yxu@jiangnan.edu.cn; wuq@jiangnan.edu.cn

Table S1 Genomic features of *B. amyloliquefaciens* MT45 compared with other 17 *B. amyloliquefaciens* strains.

**Strain number**	**Size (Mb)**	**GC%**	**Accession number**	**Genes**	**Proteins**
DSM7^T	3.9802	46.1	FN597644.1	4030	3811
TA208	3.93751	45.8	CP002627.1	3974	3847
LL3	4.00199	45.7	CP002634.1	4037	3887
XH7	3.9392	45.8	CP002927.1	3983	3846
IT-45	3.93687	46.6	CP004065.1	3832	3678
Y2	4.23862	45.9	CP003332.1	4148	3983
CC178	3.91683	46.5	CP006845.1	3795	3641
LFB112	3.94275	46.7	CP006952.1	3801	3637
L-H15	3.90597	46.7	CP010556.1	3769	3615
KHG19	3.95336	46.6	CP007242.1	3816	3658
L-S60	3.90302	46.7	CP011278.1	3773	3611
MBE1283	3.97993	46.5	CP013727.1	3856	3681
S499	3.93593	46.6	CP014700.1	3819	3671
UMAF6639	4.03464	46.3	CP006058.1	3879	3716
UMAF6614	4.00514	46.5	CP006960.1	3850	3695
B15	4.00675	46.5	CP014783.1	3875	3704
RD7-7	3.68821	46.3	CP016913.1	3656	3483
MT45	3.89752	46.1	CP011252	3873	3691

Table S2 Comparison of fold-changes of differentially expressed genes obtained by Illumina RNA-Seq and real-time PCR. The fold changes relative to 12 h revealed by real-time PCR of the selected genes were determined based on the threshold cycle (Ct) values and $2^{-\Delta\Delta CT}$ method. Three replicates were performed for each gene.

**Genes in MT45**	**24 h**		**36h**	
	**RNA-Seq**	**real-time PCR**	**RNA-Seq**	**real-time PCR**
srfAA	0.22	0.16 ± 0.08	1.88	2.52 ± 0.22
comA	0.57	0.62 ± 0.12	1.21	1.62 ± 0.34
sigA	0.52	0.33 ± 0.15	1.18	1.55 ± 0.02
degU	0.37	0.18 ± 0.11	1.59	1.26 ± 0.15
abrB	0.12	0.15 ± 0.08	1.03	1.55 ± 0.23
rghR	0.28	0.35 ± 0.14	2.52	2.96 ± 0.34
rapC	6.74	8.76 ± 0.38	0.27	0.35 ± 0.05
rapH1	53.35	12.22 ± 0.53	0.15	0.27 ± 0.11
rapH3	6.33	5.85 ± 0.23	0.41	0.65 ± 0.24
rapF	2.27	3.22 ± 0.09	0.34	0.48 ± 0.14
codY	1.23	2.32 ± 0.15	0.50	0.34 ± 0.17
perR	1.62	2.54 ± 0.18	0.67	0.78 ± 0.23
sinI	2.94	3.16 ± 0.25	0.38	0.21 ± 0.06
spx	1.90	2.84 ± 0.16	0.51	0.65 ± 0.24
phoP	1.64	3.05 ± 0.52	0.38	0.52 ± 0.16
**Genes in DSM7^{T}**				
srfAA	0.94	0.76 ± 0.12	0.86	1.24 ± 0.37
comA	1.96	2.87 ± 0.65	2.60	1.59 ± 0.48
sigA	1.09	0.98 ± 0.13	0.89	1.35 ± 0.21
degU	0.91	1.23 ± 0.14	1.09	0.87 ± 0.19
abrB	0.43	0.66 ± 0.12	0.29	0.11 ± 0.05
rghR	0.64	0.81 ± 0.32	0.66	0.47 ± 0.13
rapC	1.26	0.96 ± 0.24	1.87	2.35 ± 0.28
*rapH*1	1.43	1.68 ± 0.26	4.47	2.86 ± 0.17
*rapH*3	1.67	1.83 ± 0.35	3.61	4.57 ± 0.41
rapF	1.35	2.02 ± 0.46	2.19	1.89 ± 0.27

codY	0.86	0.65 ± 0.34	0.68	0.97 ± 0.25
perR	3.60	5.56 ± 0.55	4.29	3.18 ± 0.13
sinI	1.70	1.25 ± 0.28	3.72	5.53 ± 0.34
spx	1.62	2.14 ± 0.36	2.63	1.99 ± 0.25
phoP	0.72	0.58 ± 0.16	0.82	1.02 ± 0.16

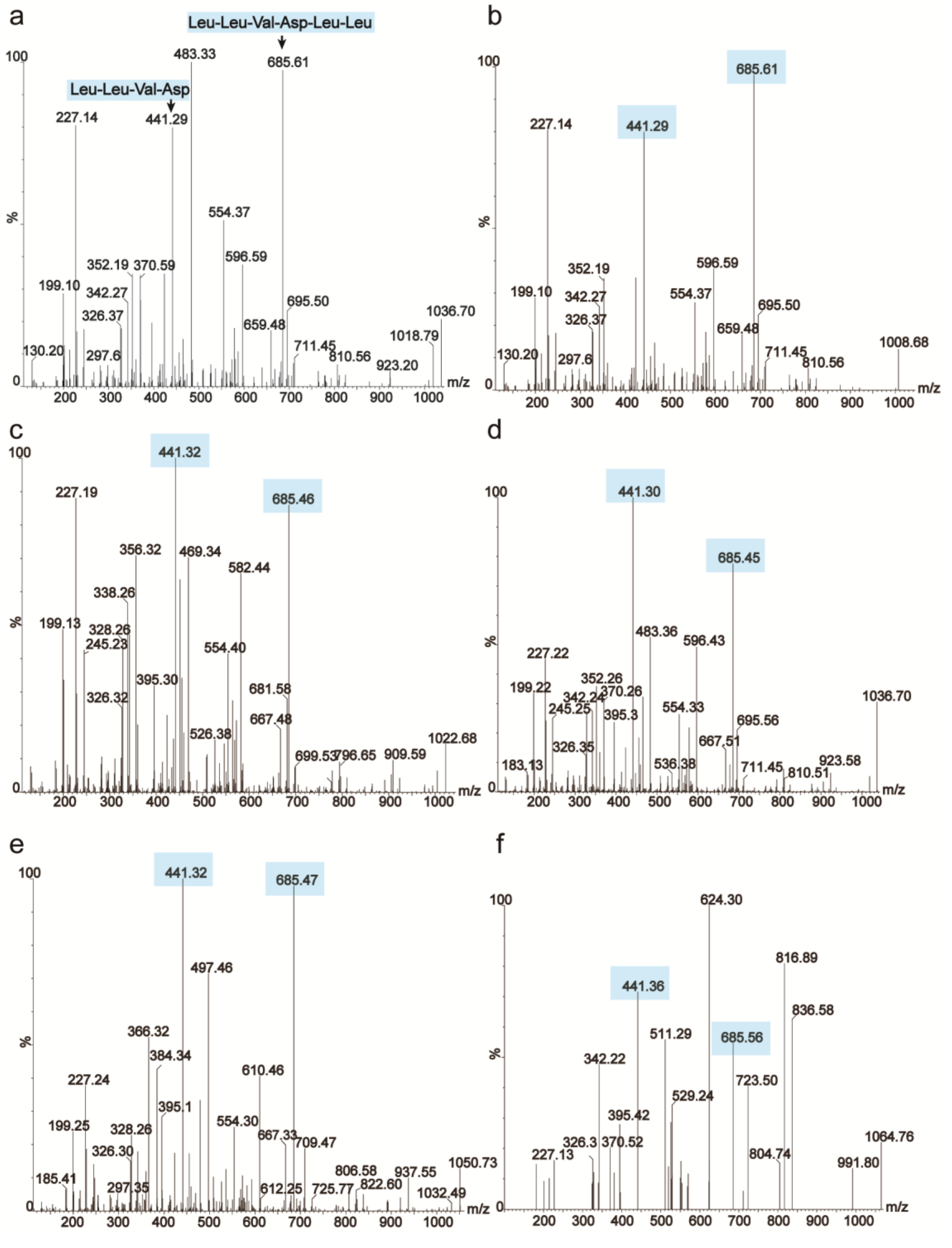

Figure S1 MS/MS spectrum of surfactin (a) MS/MS spectra of protonated cyclic standard C15 surfactin ions at m/z 1036.70; (b)-(f) MS/MS spectra of protonated ions of C13, C14, C15, C16, C17 surfactin homologues produced by MT45 at m/z 1008.6, 1022.68, 1036.70, 1050.73, and 1064.78.

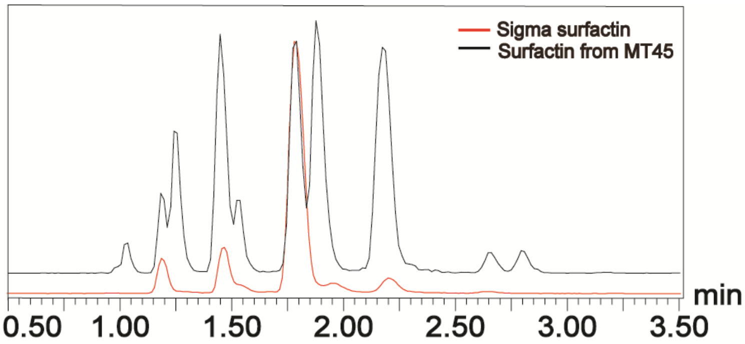

Figure S2 UPLC chromatogram of Sigma surfactin and surfactin from MT45. The chromatographic separation was performed on a Waters Acquity C18 column (50 mm × 2.1 mm, 1.7μm particle). The mobile phase consisted of solvent A (HPLC grade water containing 0.1% formic acid) and solvent B (HPLC grade methanol). Elution was performed by linear biphasic gradient of 85–100% solvent B over 6 min, at a flow rate of 0.3 mL/min.

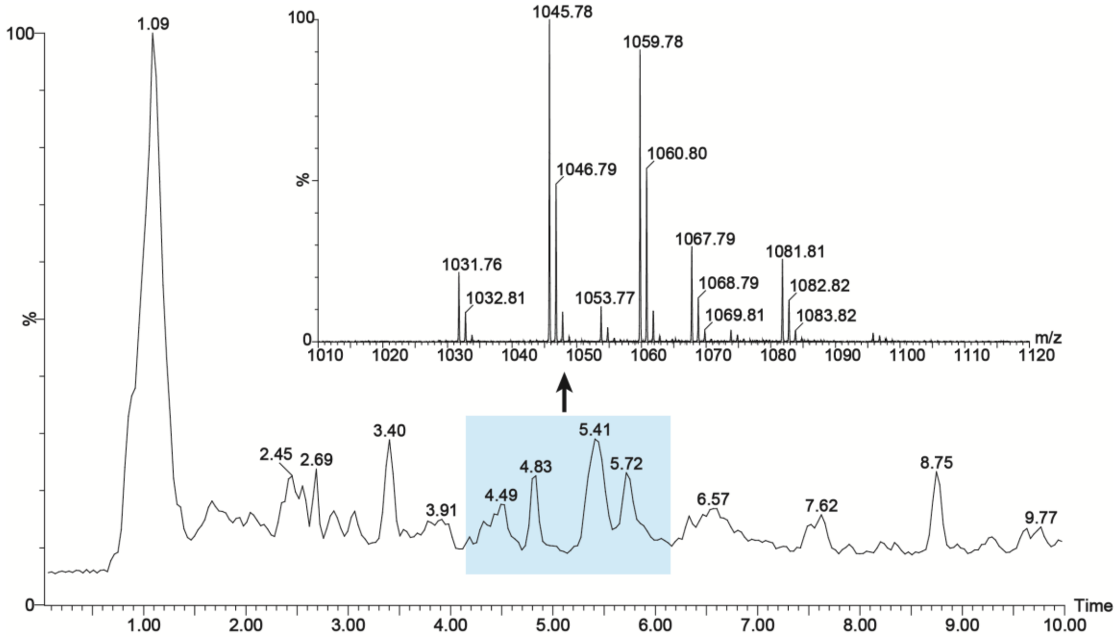

Figure S3 Detection of iturin family lipopeptide bacillomycin D by LC-MS.

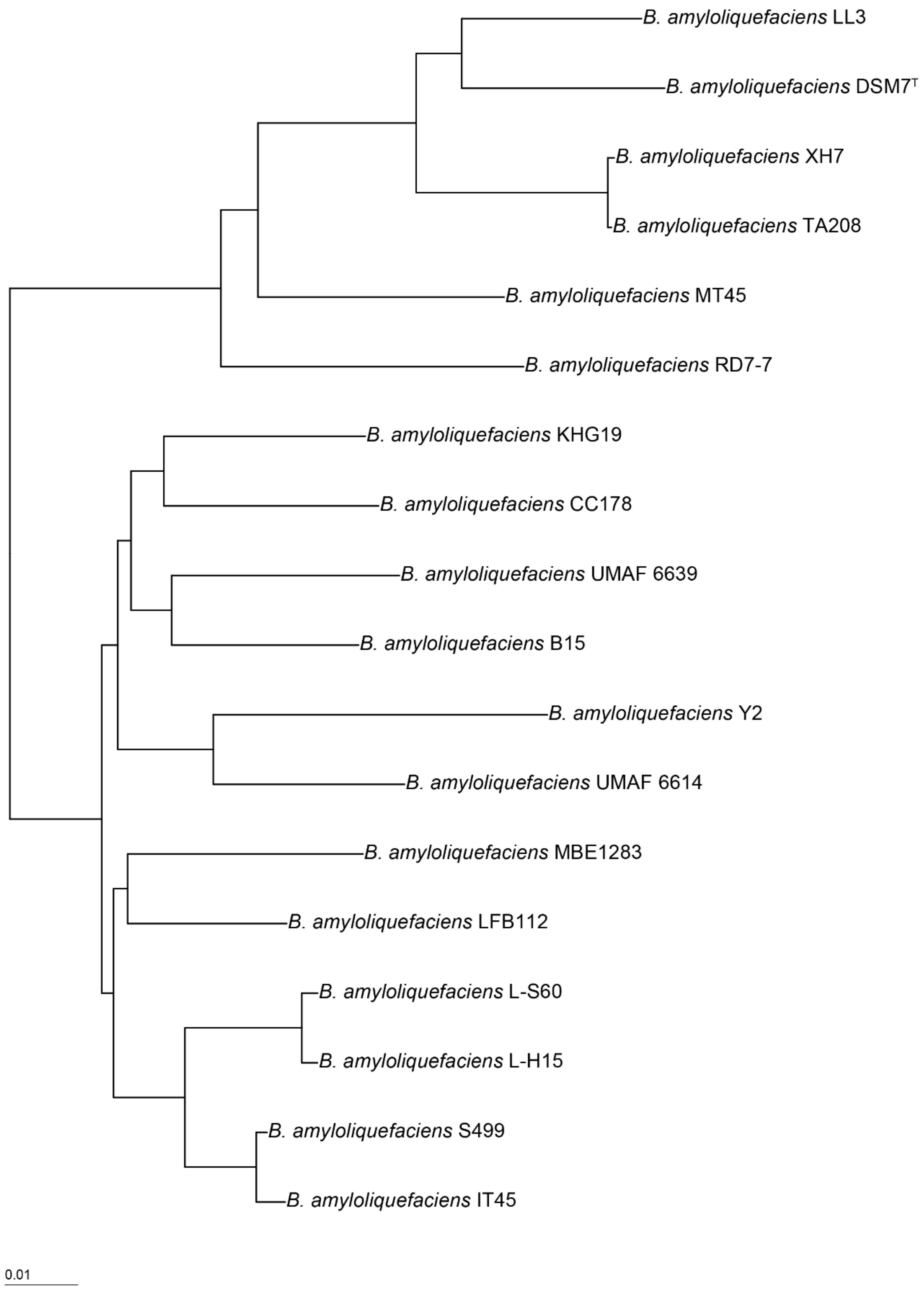

Figure S4 Phylogenetic tree generated via comparison of the genome of *B. amyloliquefaciens* MT45 with other completely sequenced genomes of *B. amyloliquefaciens* deposited in NCBI.

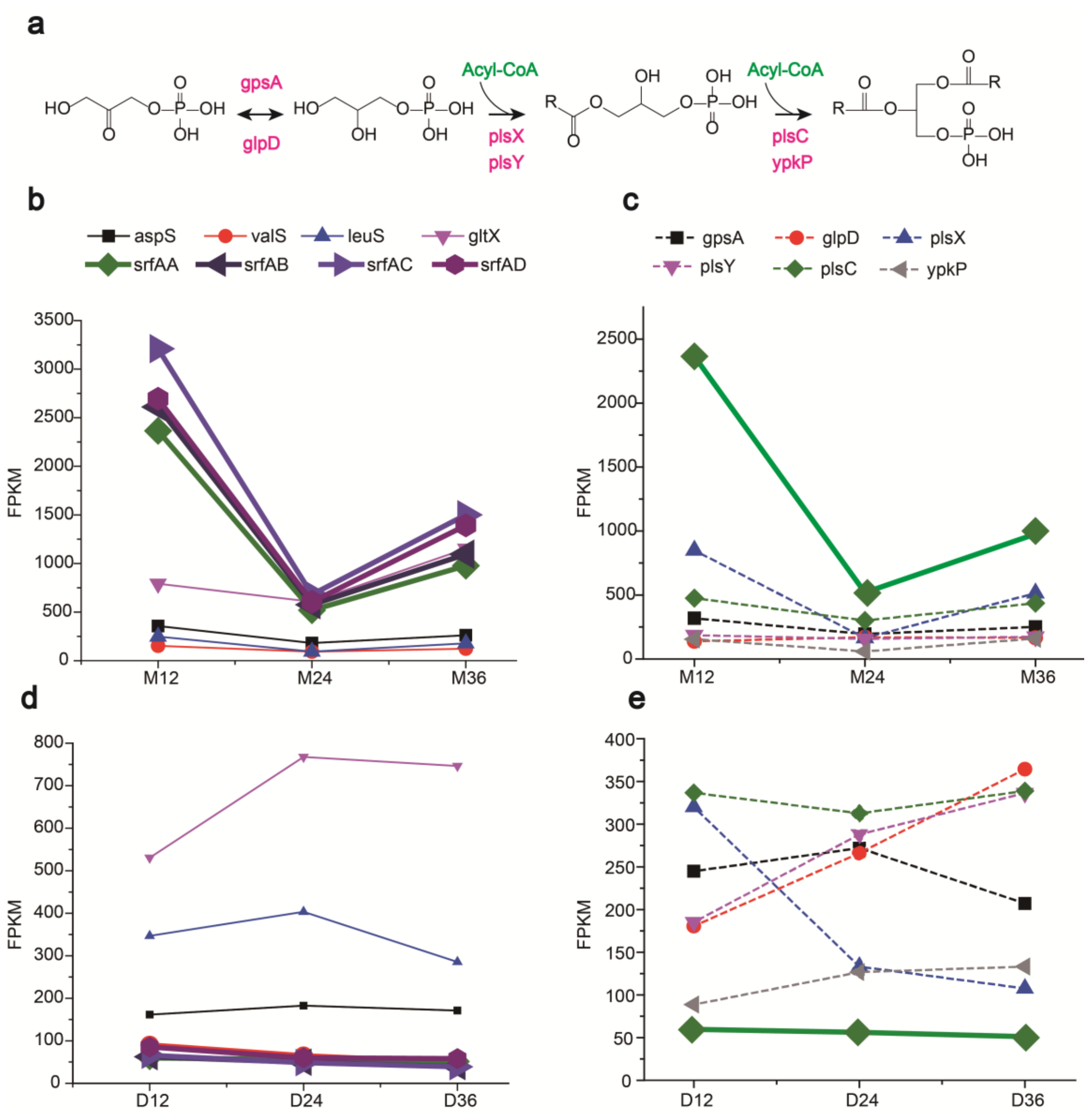

Figure S5 Competitive utilization of amino acids and fatty acids for surfactin and biomass biosynthesis in MT45 and DSM7^{T}. (a) Scheme and genes of the biosynthesis of phospholipid from fatty acids. (b) and (d) Transcriptional comparison of aminoacyl-tRNA synthetase including *aspS*, *valS*, *leuS* and *gltX* with the *srfA* operon at different growth stage in MT45 and DSM7^{T} respectively. (c) and (e) Transcriptional comparison of genes involved in phospholipid biosynthesis with *srfAA* gene at different growth stage MT45 and DSM7^{T} respectively.

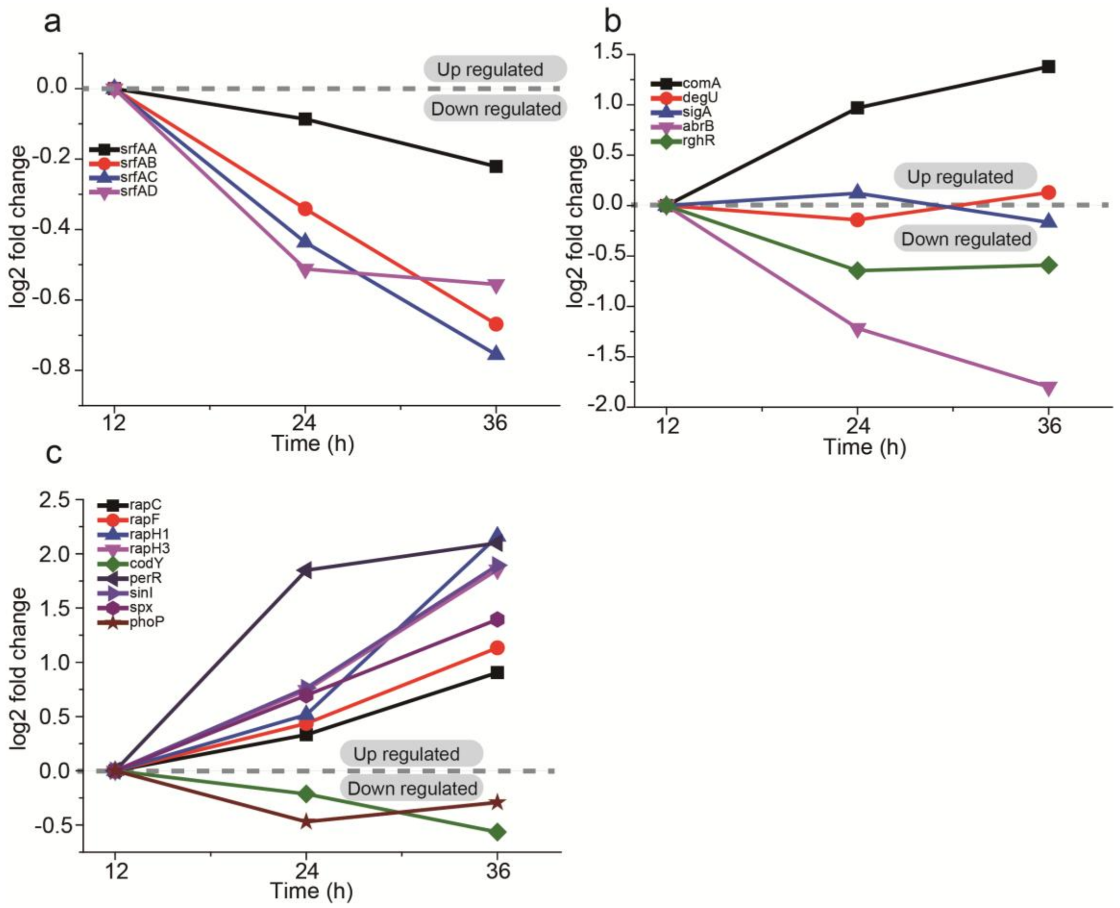

Figure S6 Expression tendency of *srfA* operon and potential regulators in DSM7^T. (a) Expression tendency of *srfA*. (b) Expression tendency of potentially positive regulators. (c) Expression tendency of potentially negative regulators. The relative expression level was exhibited as log2 fold change to 12 h.
